# Ultrasound Imaging of the Superficial and Deep Fasciae Thickness of Upper Limbs in Lymphedema Patients Versus Healthy Subjects

**DOI:** 10.3390/diagnostics14232697

**Published:** 2024-11-29

**Authors:** Carmelo Pirri, Nina Pirri, Chiara Ferraretto, Lara Bonaldo, Raffaele De Caro, Stefano Masiero, Carla Stecco

**Affiliations:** 1 Department of Neurosciences, Institute of Human Anatomy, University of Padova, 35121 Padova, Italy; rdecaro@unipd.it (R.D.C.); carla.stecco@unipd.it (C.S.); 2 Department of Medicine—DIMED, School of Radiology, Radiology Institute, University of Padova, 35122 Padova, Italy; nina_92_@hotmail.it; 3 Physical Medicine and Rehabilitation School, University of Padova, 35128 Padova, Italy; chiara.ferraretto@gmail.com; 4 Department of Neuroscience, Section of Rehabilitation, University of Padova, 35121 Padova, Italy; lara.bonaldo@aopd.veneto.it (L.B.); stef.masiero@unipd.it (S.M.)

**Keywords:** lymphedema, superficial fascia, deep fascia, cutis, dermis, subcutaneous tissue, ultrasound imaging, skin, thickness, ultrasound imaging, ultrasonography

## Abstract

Background/Objectives: Lymphedema, a common source of disability among oncology patients, necessitates continuous targeted rehabilitation. Recent studies have revealed the role of connective tissue in this pathology; however, despite existing research on ultrasound (US) use in lymphedema, no studies have specifically addressed the use of ultrasound to assess fasciae in patients with lymphedema. This study aims to provide a more objective characterization of typical US alterations in these patients by quantifying the thickness of superficial and deep fasciae and comparing them with those of healthy volunteers. Methods: A cross-sectional study was performed using US imaging to measure the thickness of superficial and deep fascia in different regions and levels of the arm and forearm in a sample of 50 subjects: 25 chronic lymphedema patients and 25 healthy participants. Results: No significant difference in fascial thickness was observed between affected and unaffected upper limbs, but patients had notably thinner superficial fascia and deep fascia compared with healthy volunteers. The findings for superficial and deep fascia revealed statistically significant differences (*p* < 0.0001) in all regions and levels. Conclusions: This study demonstrates the effectiveness of US imaging as a non-invasive tool for detecting subtle fascial changes in chronic lymphedema patients, revealing thinner fasciae compared with those in healthy volunteers. These findings suggest a potential anatomical predisposition to lymphedema, highlighting the importance of incorporating detailed US assessments in diagnosis and management to improve early intervention and patient outcomes. Future studies could, therefore, investigate whether preventive fascia assessment might improve the early identification of individuals at risk.

## 1. Introduction

Lymphedema is a chronic condition characterized by the accumulation of protein-rich interstitial fluid resulting from impaired lymphatic drainage [1]. This pathology can be classified as either primary, due to congenital malformations of the lymphatic system, or secondary, arising from surgical interventions, trauma or infections, as often observed following breast cancer treatments. Secondary lymphedema is frequently seen in patients undergoing axillary lymph node dissection and postoperative radiotherapy, with significant impacts on the quality of life, including reduced limb function, chronic pain, recurrent infections and a deterioration in psychological well-being [1,2,3]. The lymphatic system plays a critical role in maintaining fluid homeostasis, immune function and the removal of metabolic waste products [4]. Dysfunction in this system, as seen in lymphedema, results in the disruption of these processes, leading to chronic inflammation, tissue remodeling and immune dysregulation. Understanding the complex pathophysiology of lymphedema is crucial for developing effective treatment strategies that address not only the physical manifestations but also the underlying molecular and cellular changes contributing to disease progression [5,6,7,8,9]. The condition often leads to progressive fibrosis and adipose tissue deposition, further complicating its management and exacerbating symptoms over time [1]. Fibrotic alterations are a key element in defining the staging and prognosis of lymphedema, yet the organization of the fibrous subcutaneous tissue and its potential variations in lymphedema have not been thoroughly investigated. Subcutaneous tissue is divided by the superficial fascia into two distinct compartments, each with unique characteristics: the superficial adipose tissue (SAT) and the deep adipose tissue (DAT) [10]. The superficial fascia is recognized as a specific anatomical structure with distinctive cellular and innervation properties. It is a thin, fibrous membrane that extends continuously throughout the body and is composed of irregularly arranged collagen fibers interspersed with numerous elastic fibers [11]. This fibrous membrane functions as a supportive scaffold for adipose lobules, providing structural integrity to the surrounding tissues. The deep fascia is a thicker fibrous structure below the DAT that envelops the muscle [11]. Given the close correlation between fascial structures, particularly the superficial fascia and the lymphatic system, it is essential to explore how these structures might change in chronic lymphedema.

In recent years, high-resolution ultrasound (US) imaging has been increasingly employed to assess tissue alterations in patients with lymphedema, as traditional imaging techniques are often costly and time-consuming [12,13]. Ultrasound offers several advantages, including low cost, portability of equipment and safety during examination. US imaging aids in supporting diagnostic hypotheses and monitoring tissue responses to decongestive therapy. Recent studies reported the potential of US imaging for the mapping and characterization of lymphedema, enhancing our understanding of the disease’s pathophysiology and supporting the development of more targeted therapeutic protocols [12,13]. US imaging is valuable due to its non-invasive nature, cost-effectiveness and ability to provide detailed anatomical and functional information, which is crucial for monitoring disease progression and the response to therapy. A comprehensive understanding of lymphatic anatomy, combined with advanced imaging techniques, is essential for accurate diagnoses and for guiding both conservative and surgical management options. Although fibrotic alterations are crucial in determining the staging and prognosis of lymphedema, the specific involvement of fibrous components such as superficial and deep fasciae have not yet been fully explored. Considering the strong correlation between fascial structures, particularly the superficial fascia and the lymphatic system, we investigated the differences between patients with chronic lymphedema and healthy subjects. In this context, the aim of this study was to assess the thickness of the superficial and deep fasciae across different levels and regions of the upper limbs.

## 2. Materials and Methods

### 2.1. Study Design

A cross-sectional observational study based on the Strengthening the Reporting of Observational Studies in Epidemiology (STROBE) statement was conducted [14] with the aim of comparing the US thickness of the superficial and deep fasciae in the lymphedematous limb with those of the healthy contralateral limb. Additionally, the US thickness of superficial and deep fasciae was analyzed and compared with that in healthy volunteers. The Helsinki Declaration and human experimentation rules [15] were considered, and the Ethics Committee of University of Padova approved the research. All participants were informed and provided written consent prior to inclusion in the study.

### 2.2. Participants

A total sample of 50 subjects was recruited from March 2023 to September 2024 and divided into two groups: “group 1” comprised 25 subjects with upper limb chronic lymphedema, and “group 2” comprised 25 healthy subjects. Based on the following criteria, the inclusion criteria for group 1 participation consisted of the following parameters: (1) Diagnosis of secondary lymphedema—typically resulting from cancer treatments, such as surgery (e.g., lymph node dissection) or radiation therapy; (2) A chronic condition—the lymphedema had to be in a chronic phase, often defined as persisting for at least 6 months; this ensured that acute or transient cases were excluded; (3) Patients were included if their lymphedema was at stage II or higher; (4) A stable condition—candidates had to have a stable limb volume, meaning that the size of the affected limb could not have fluctuated by more than 10% over the last two years; this was important for ensuring that the US imaging was not confounded by sudden changes in limb size. Patients with lymphedema affecting the lower limbs, primary lymphedema, or lymphedema caused by venous insufficiency were excluded from consideration, as were those presenting with bilateral lymphedema. Additionally, individuals with active lymphangitis, male patients and those whose edema was linked to systemic conditions such as heart, liver or kidney failure were not included in the study. The healthy volunteers in group 2 were matched with the lymphedema patients in terms of age, sex and BMI. The participants underwent a US examination to assess their US fascial thickness. The enrollment of subjects was performed by a specialized medical doctor with more than 8 years of experience in physical and rehabilitation medicine.

### 2.3. Ultrasound Examination Measurements

Ultrasound examinations were conducted using two high-resolution US systems (Hitachi Avius, Hitachi, Milan, Italy, and Edge II, Sonosite, FUJIFILM, Inc., Bothell, WA, USA), which were equipped, respectively, with an L75 linear probe with a frequency range of 5–18 MHz and a linear probe with a frequency range of 6–15 MHz. The assessments were performed by a physician specializing in physical and rehabilitation medicine who possessed eight years of expertise in the ultrasound examination of fasciae (C.P.). The US system was configured to operate at a standard sound velocity of c = 1540 m/s, a typical setting for diagnostic ultrasounds and was used in the B-mode with a penetration depth of 30 mm. A generous layer of US gel was applied to ensure optimal probe–skin contact while minimizing pressure on the skin. The probe was placed gently to avoid tissue compression and oriented perpendicularly to the fascial layers to mitigate anisotropy artifacts. Both the power and gain settings were standardized and consistently applied across all the evaluations to maintain uniform image quality and diagnostic accuracy. A short-axis view was adopted to facilitate optimal visualization and tracking of anatomical landmarks associated with the fascial layers, providing better continuity and differentiation of these structures throughout the examination. Identical scanning techniques were applied to both the affected and contralateral healthy limb for each patient and healthy volunteer. During the procedure, patients and healthy volunteers were positioned supine for the anterior regions and prone for the posterior regions, with their limbs resting comfortably on the examination table. For each level and region assessed, the protocol outlined by Pirri et al. [16,17] was followed:

Arm:
  - Anterior region: The patient was positioned supine with the arm in a neutral position.
    ◦ Anterior (Ant1): The probe was positioned in a short-axis view over the proximal half of the arm. The median nerve and brachial artery were located medially, between the brachialis and biceps muscles.
    ◦ Anterior 2 (Ant 2): The probe was moved distally, just above the cubital fossa, following the median nerve and brachial artery. The brachialis muscle was visualized deep within the biceps muscle.
  - Posterior region: The patient was positioned prone, with the arm in a neutral position.
    ◦ Posterior 1 (Post 1): The probe was placed in a short-axis view over the proximal half of the posterior arm, allowing visualization of the tricep muscle heads, along with the median, ulnar and radial nerves.
    ◦ Posterior 2 (Post 2): The probe was moved distally to just above the elbow, following the anatomical landmarks.
Forearm:
  - Anterior region: The patient was positioned supine, with the arm in a neutral position and the forearm supinated.
    ◦ Anterior 1 (Ant 1): The probe was positioned in a short-axis view over the proximal anterior forearm. The pronator teres muscle was lateral to the flexor carpi radialis (FCR) and flexor digitorum superficialis (FDS). The median nerve was located between the two heads of the pronator teres.
    ◦ Anterior 2 (Ant 2): The probe was moved distally to the anterior forearm. The flexor digitorum profundus (FDP) was visualized deep within the FDS, flexor carpi ulnaris (FCU) and palmaris longus. These muscles were located above the interosseous membrane.
  - Posterior region: The patient was positioned prone, with the arm in a neutral position and the forearm pronated.
    ◦ Posterior 1 (Post 1): The probe was placed in a short-axis view over the proximal posterior forearm, just below the elbow. The extensor digitorum (ED) muscle was located between the extensor carpi radialis brevis (ECRB) and extensor digitorum brevis (EDB), above the supinator muscle. Medially, the brachioradialis, along with the radial artery and nerve branches, was visualized.
    ◦ Posterior 2 (Post 2): The probe was moved distally along the posterior forearm. The ED muscle decreased in size, while the EDB became more prominent. The abductor pollicis longus (APL) was found near the radius, with the extensor pollicis longus (EPL) close to the ulna. The extensor carpi ulnaris (ECU) was positioned medially, above the ulna and adjacent to the EDB, EPL and APL. The extensor pollicis brevis and extensor indicis muscles were also visualized.

Upon identifying the relevant anatomical landmarks, including bones, muscles, nerves and tendons for each point, the image depth was adjusted to optimize visualization of the superficial layers. The focus was generally placed between 0.5 and 2 cm to provide clear imaging within the first 3 cm of depth. However, for the posterior arm, the focus was extended to 3 cm to accommodate the deeper structures in these regions, ensuring consistent image clarity across all the regions and levels examined.

### 2.4. Ultrasound Image Analysis

Ultrasound images were captured and saved at the end of each evaluation session. For each level and region, the thickness of the superficial and deep fasciae was measured in millimeters using ImageJ software (available online at: https://imagej.nih.gov/iJ/; accessed on 5 September 2024). To mitigate potential variations in thickness, each image was divided into three sections, and within each section, into three locations with optimal visibility within each region, and the average value was used for statistical analysis. The superficial thickness was measured at the undulating hyperechoic structure that traverses the subcutaneous tissue, while the deep fascia thickness was determined at the hyperechoic interface between the subcutaneous tissue and the underlying muscle.

### 2.5. Statistical Analysis

Statistical analysis was conducted using GraphPad PRISM version 8.4.2 (GraphPad Software Inc., San Diego, CA, USA), considering *p* < 0.05 as the threshold for statistical significance. Effect sizes were computed using G*Power 3.1 (Universität Düsseldorf, Psychology Department) and interpreted in accordance with Cohen’s guidelines: small (d = 0.20), medium (d = 0.50) and large (d = 0.80) [18]. In our previous research [11], the effect size for the superficial fascia of the arm and forearm was found to be d = 1.2, which has been supported by subsequent studies [16]. Statistical parameters included an error probability (α error prob) of 0.05 and a statistical power (1-β error prob) of 0.95, resulting in a calculated requirement of 10 participants for adequate power. Nevertheless, we were able to extend our study cohort, ultimately enrolling 50 patients, 25 for each group, which strengthened the robustness and validity of our analysis. To assess data normality, the Kolmogorov–Smirnov test was employed. Descriptive statistics—including measures of central tendency and dispersion—were reported as the mean and standard deviation (SD) for parametric data. Comparative analysis across multiple levels was performed using one-way analysis of variance (ANOVA), followed by Tukey’s post hoc test to assess pairwise differences. For comparisons between the pathological and contralateral healthy limbs, a paired Student’s t-test was used, while for comparisons between patients and healthy volunteers, an unpaired Student’s t-test was used. Moreover, a type C two-way mixed-model intra-class correlation coefficient (ICC_3,k_) was used to assess the intra-rater reliability. The ICC values were interpreted as poor when they were below 0.5, moderate when they were between 0.5 and 0.75, good when they were between 0.75 and 0.90 and excellent when they were above 0.90 [19].

## 3. Results

### 3.1. Descriptive Data

A total of 50 subjects (50 females) participated in this study. The descriptive data of the sample are summarized in Table 1. No differences were present for the BMI, height, weight and age, showing homogeneity among the groups.

Of the Group 1 participants, 23 had been diagnosed with breast carcinoma, while 2 had scapular melanoma. The interval from their initial surgery varied from 3 to 26 years at the time of evaluation, with 75.6% having undergone further surgical procedures beyond the initial surgery. Regarding the type of surgery, 50% of the patients had undergone a mastectomy, 39.7% had had a quadrantectomy, and 10.35% had had a wide-margin excision performed for scapular melanoma. Furthermore, 94.8% of the participants had an axillary lymph node dissection, whereas only 7.1% had undergone sentinel lymph node biopsy.

### 3.2. Ultrasound Thickness Measurements

#### 3.2.1. Superficial Fascia of the Arm in Chronic Lymphedema Patients (Group 1)

Regarding Table 2, in the chronic lymphedema patients, the superficial fascia of the arm had a mean US thickness of 0.29 ± 0.06 mm (Table 2). No level or region exhibited a statistically significant difference in thickness between affected and healthy limbs.

#### 3.2.2. Superficial Fascia of the Arm in Healthy Volunteers (Group 2)

In the healthy volunteers, the superficial fascia of the arm in the different regions and levels had a mean US thickness of 0.45 ± 0.09 mm (Table 3). No level or region exhibited a statistically significant difference in thickness between affected and healthy limbs.

#### 3.2.3. Ultrasound Measurements of the Arm Superficial Fascia: Comparison Between Group 1 and Group 2

**Figure 1:**
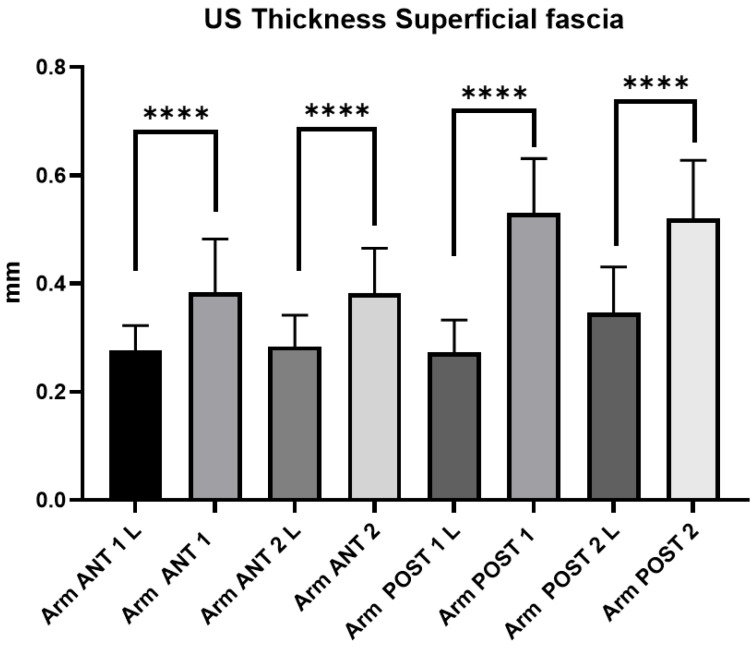
Comparison between group 1 (chronic lymphedema patients) and 2 (healthy volunteers): Ultrasound thickness measurements of the superficial fascia at different regions and levels of the arm. Abbreviations: L = lymphedema. ****: <0.0001.

The comparison between group 1 and group 2 showed statistically significant differences in the US thickness of superficial fasciae from all regions and levels (*p* < 0.0001). The US thickness of the superficial fascia of the arm in chronic lymphedema patients (group 1) is thinner (mean: 0.29 ± 0.06 mm) than that in healthy volunteers (group 2) (mean: 0.45 ± 0.09 mm) (Figure 1).

#### 3.2.4. Superficial Fascia of the Forearm in Chronic Lymphedema Patients (Group 1)

Regarding Table 4, in the chronic lymphedema patients, the superficial fascia of the forearm had a mean US thickness of 0.28 ± 0.04 mm (Table 4). No level or region exhibited a statistically significant difference in thickness between affected and healthy limbs.

#### 3.2.5. Superficial Fascia of the Forearm in Healthy Volunteers (Group 2)

In the healthy volunteers, the superficial fascia of the forearm in the different regions and levels had a mean US thickness of 0.39 ± 0.09 mm (Table 5). No level or region exhibited a statistically significant difference in thickness between affected and healthy limbs.

#### 3.2.6. Ultrasound Measurements of the Forearm Superficial Fascia: Comparison Between Group 1 and Group 2

**Figure 2:**
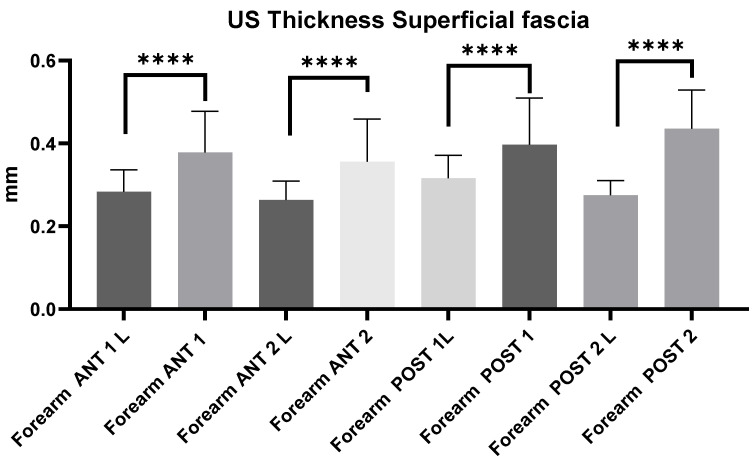
Comparison between group 1 (chronic lymphedema patients) and 2 (healthy volunteers): Ultrasound thickness measurements of the superficial fascia at different regions and levels of the forearm. Abbreviations: L = lymphedema. ****: <0.0001.

The comparison between group 1 and group 2 showed statistically significant differences in the US thickness of the superficial fasciae from all regions and levels (*p* < 0.0001). The US thickness of the superficial fascia of the forearm in chronic lymphedema patients (group 1) is thinner (mean: 0.28 ± 0.04 mm) than that in healthy volunteers (group 2) (mean: 0.39 ± 0.09 mm) (Figure 2).

#### 3.2.7. Deep Fascia of the Arm in Chronic Lymphedema Patients (Group 1)

Regarding Table 6, in the chronic lymphedema patients, the deep fascia of the arm had a mean US thickness of 0.46 ± 0.1 mm (Table 6). No level or region exhibited a statistically significant difference in thickness between affected and healthy limbs.

#### 3.2.8. Deep Fascia of the Arm in Healthy Volunteers (Group 2)

In the healthy volunteers, the superficial fascia of the forearm in the different regions and levels had a mean US thickness of 0.71 ± 0.13 mm (Table 7). No level or region exhibited a statistically significant difference in thickness between affected and healthy limbs.

#### 3.2.9. Ultrasound Measurements of Arm Deep Fascia: Comparison Between Group 1 and Group 2

**Figure 3:**
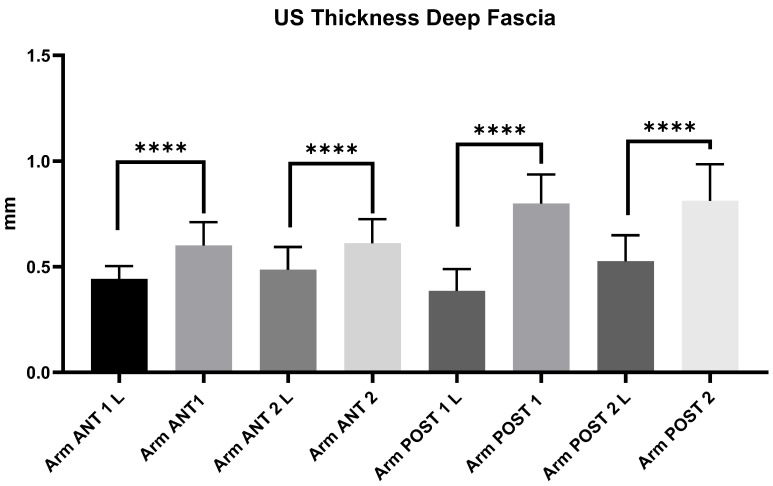
Ultrasound thickness measurements of the deep fascia at the different regions and levels of the arm. Abbreviations: L = lymphedema. ****: <0.0001.

The comparison between group 1 and group 2 showed statistically significant differences in the US thickness of deep fascia of all regions and levels (*p* < 0.0001). The US thickness of superficial fascia of the forearm in chronic lymphedema patients (group 1) is thinner (mean: 0.46 ± 0.1 mm) than that in healthy volunteers (group 2) (mean: 0.71 ± 0.13 mm) (Figure 3).

#### 3.2.10. Deep Fascia of the Forearm in Chronic Lymphedema Patients (Group 1)

Regarding Table 8, in the chronic lymphedema patients, the deep fascia of the arm had a mean US thickness of 0.46 ± 0.11 mm (Table 6). No level or region exhibited a statistically significant difference in thickness between affected and healthy limbs.

#### 3.2.11. Deep Fascia of the Forearm in Healthy Volunteers (Group 2)

In the healthy volunteers, the superficial fascia of the forearm in the different regions and levels had a mean US thickness of 0.71 ± 0.13 mm (Table 9). No level or region exhibited a statistically significant difference in thickness between affected and healthy limbs.

#### 3.2.12. Ultrasound Measurements of Forearm Deep Fascia: Comparison Between Group 1 and Group 2

**Figure 4:**
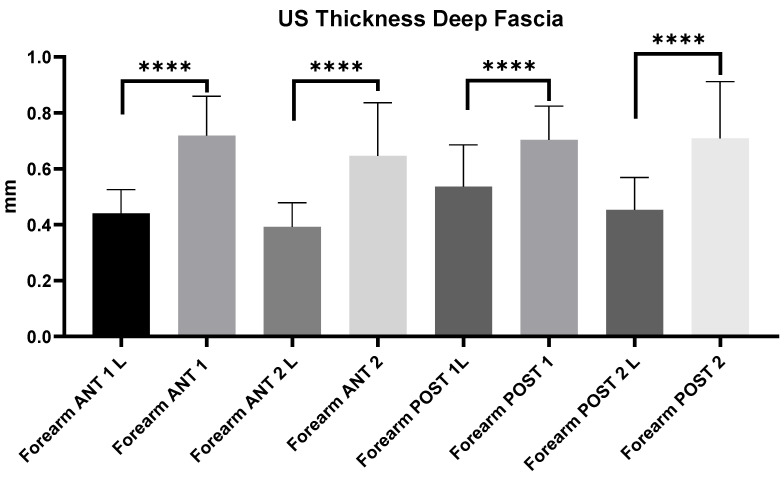
Ultrasound thickness measurements of the deep fascia at different regions and levels of the forearm. Abbreviations: L = lymphedema. ****: <0.0001.

The comparison between group 1 and group 2 showed statistically significant differences in the US thickness of the deep fascia at all regions and levels of the forearm (*p* < 0.0001). The US thickness of the superficial fascia of the forearm in chronic lymphedema patients (group 1) is thinner (mean: 0.46 ± 0.11 mm) than that in healthy volunteers (group 2) (mean: 0.71 ± 0.13 mm) (Figure 4).

### 3.3. Correlation Between Ultrasound Measurements and the Descriptive Data

According to the correlation analysis, there was a statistical correlation between the thickness of the forearm superficial fascia and the BMI for the Ant 2 region in the chronic lymphedema patients (r = −0.5135; *p* = 0.0001) and between the forearm deep fascia and age for the Post 1 region in the chronic lymphedema patients (r = −0.2831; *p* = 0.0463).

### 3.4. Intra-Rater Reliability

In addition, the intra-rater reliability was reported as being good and excellent. The results were as follows: for superficial fasciae of the arm: group 1, chronic lymphedema (ICC_3,k_: 0.90; 0.85–0.95) vs. group 2, healthy (ICC_3,k_: 0.91; 0.88–0.94); for superficial fascia of the forearm: group 1, chronic lymphedema (ICC_3,k_: 0.89; 0.84–0.94) vs. group 2, healthy (ICC_3,k_: 0.91; 0.88–0.94); for deep fascia of the arm: group 1, chronic lymphedema (ICC_3,k_: 0.90; 0.85–0.95) vs. group 2, healthy (ICC_3,k_: 0.90; 0.85–0.95); for deep fascia of the forearm: group 1, chronic lymphedema (ICC_3,k_: 0.90; 0.85–0.95) vs. group 2, healthy (ICC_3,k_: 0.90; 0.85–0.95).

## 4. Discussion

To the best of our current knowledge, this study may be stated as the first study to detail the fasciae thickness in the arm and forearm in chronic lymphedema patients compared with healthy volunteers. As has been reported by other studies examining superficial and deep fasciae by US imaging, the superficial fasciae appeared as a hyperechoic undulating layer identified within the subcutaneous tissue, positioned beneath the (epi)dermis, that partitions the subcutaneous tissue into two compartments: the superficial adipose tissue (SAT) and the deep adipose tissue (DAT); in contrast, the deep fascia appeared as a linear hyperechoic layer below the subcutaneous tissue that surrounds the muscles [16,17].

**Figure 5:**
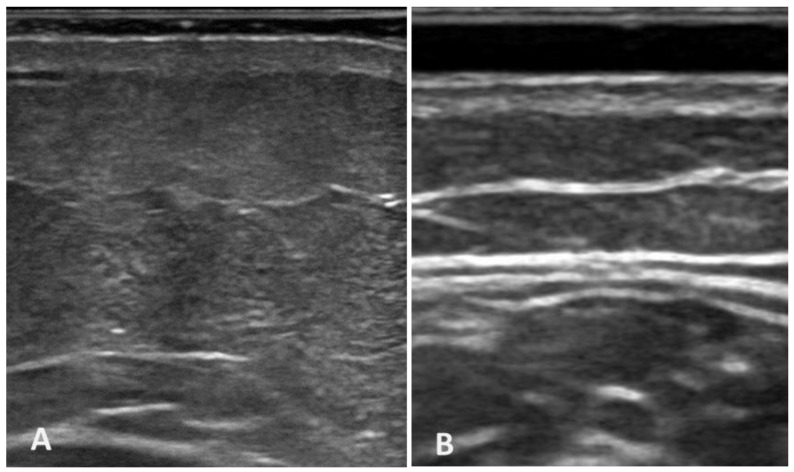
**Ultrasound imaging of superficial and deep fasciae in chronic lymphedema patients and healthy volunteers.** (**A**) Ultrasound image of chronic lymphedema, displaying thin superficial and deep fasciae. This reduced thickness is characteristic of chronic lymphatic stasis, which is likely due to tissue remodeling associated with prolonged lymphedema. (**B**) Ultrasound image of healthy volunteers, showing good visibility of superficial and deep fasciae, highlighting the structural integrity of the fasciae and subcutaneous tissue.

The study’s primary aim was to investigate the difference in the superficial and deep fascia of the arm and forearm in chronic lymphedema patients compared with those in healthy volunteers. An analysis of our results on the superficial fascial thickness of the arm and forearm showed that in group 1, at the anterior and posterior regions/levels, the thickness was thinner (Arm: Ant 1 = 0.28 ± 0.05; Ant 2 = 0.28 ± 0.05; Post 1 = 0.28 ± 0.06; Post 2 = 0.35 ± 0.08; Forearm: Ant 1 = 0.28 ± 0.05; Ant 2 = 0.27 ± 0.05; Post 1 = 0.31 ± 0.06; Post 2 = 0.28 ± 0.03) than in group 2 (Arm: Ant 1 = 0.38 ± 0.1; Ant 2 = 0.38 ± 0.08; Post 1 = 0.53 ± 0.1; Post 2 = 0.52 ± 0.1; Forearm: Ant 1 = 0.38 ± 0.1; Ant 2 = 0.36 ± 0.1; Post 1 = 0.40 ± 0.11; Post 2 = 0.43 ± 0.09) (Table 2, Table 3, Table 4 and Table 5; Figure 1, Figure 2 and Figure 5).

In light of these findings, the superficial fascia tends to be thinner in the chronic lymphedema patients. It remodeled over time in response to the evolution of lymphedema. Moreover, no significant difference was identified in the thickness of superficial fasciae between affected and healthy limbs in chronic lymphedema patients. These findings have important clinical implications due to the close anatomical relationship between superficial fasciae and the lymphatic system. Lymphatic vessels rely on the extracellular matrix’s stability, upheld by anchoring filaments, to maintain their structure. Consequently, disruptions to the superficial fascia and its anchoring elements—retinacula cutis—may further hamper lymphatic drainage. Moreover, the connection between lymphatic vessels and the fibrous septa within superficial adipose tissue suggests that connective tissue organization might play a role in lymphatic transport from dermal plexuses to deeper lymphatic collectors [18,19,20]. Fibrosis and reduced elasticity in the subcutaneous tissue likely compromise this drainage capacity [21]. However, this study did not find measurable differences in the superficial fascia thickness between the affected and unaffected limbs among chronic lymphedema patients, although it appears to be significantly thinner than that in healthy volunteers. This discrepancy could point to an intrinsic structural modification within the superficial fascia of lymphedema patients, raising the possibility that these individuals may possess a predisposing fascial vulnerability that could contribute to the onset or exacerbation of lymphedema. Such alterations may stem from an underlying connective tissue insufficiency or decreased resilience, which might compromise the superficial fascia’s capacity to support normal lymphatic function and transport. This inherent anatomical weakness could mean that these patients are more susceptible to fluid retention, lymphatic congestion and, consequently, the development of chronic lymphedema. Consequently, by closely evaluating the fascial structures through advanced US imaging, clinicians may better identify high-risk patients, monitor lymphedema progression and implement targeted therapeutic approaches to mitigate fascial weakening before it significantly impairs lymphatic drainage. This expanded anatomical understanding advocates for a more proactive and preventive approach in the management of chronic lymphedema.

In addition, statistically differences in the thickness of the deep fascia of the arm and forearm were evident between the two groups: Group 1—Arm: Ant 1 = 0.44 ± 0.06; Ant 2 = 0.49 ± 0.1; Post 1 = 0.39 ± 0.1; Post 2 = 0.53 ± 0.12; Forearm: Ant 1 = 0.44 ± 0.08; Ant 2 = 0.39 ± 0.09; Post 1 = 0.54 ± 0.15; Post 2 = 0.45 ± 0.11; Group 2—Arm: Ant 1 = 0.72 ± 0.14; Ant 2 = 0.65 ± 0.20; Post 1 = 0.80 ± 0.14; Post 2 = 0.81 ± 0.17; Forearm: Ant 1 = 0.6 ± 0.11; Ant 2 = 0.61 ± 0.11; Post 1 = 0.70 ± 0.12; Post 2 = 0.71 ± 0.2 (Table 6, Table 7, Table 8 and Table 9; Figure 3, Figure 4 and Figure 5). This study also noted a significant decrease in the deep fascia thickness in chronic lymphedema patients compared with healthy volunteers. Although the deep fascia did not display gross structural alterations, its reduced thickness may indicate an underlying weakness or altered biomechanical integrity. Such thinning in the deep fascia could reflect an adaptive response to prolonged edema, or alternatively, an innate connective tissue vulnerability in these patients. The deep fascia plays a critical role in compartmentalizing muscular tissue, supporting vascular and lymphatic networks that arrive at subcutaneous tissue, facilitating normal fluid dynamics within the limb [11,22,23]. A thinner deep fascia may lack the necessary tension and elasticity to provide adequate support for lymphatic and vascular structures, thus compromising lymphatic function and promoting fluid stasis. This reduction in structural resilience might not only impede lymphatic drainage but could also disrupt the reciprocal communication between the deep fascia and the overlying superficial layers, further exacerbating tissue congestion and fibrosis [24,25]. The implications of a thinner deep fascia for clinical practice are significant. By identifying patients with reduced deep fascial thickness through US imaging, clinicians can more accurately assess potential anatomical factors contributing to lymphedema progression. Such high US imaging may guide targeted interventions aimed at reinforcing fascial stability, improving tissue elasticity and enhancing lymphatic drainage. In essence, these findings highlight the necessity for comprehensive US examinations that incorporate quantitative evaluations of fasciae, paving the way for more personalized and proactive management strategies in chronic lymphedema.

Moreover, there is increasing interest in understanding the role of the retinacula cutis in the development of fibrosis within the subcutaneous tissue. The retinacula cutis, consisting of fibrous septa that anchor the skin to superficial fascia and the latter to deep fascia, may contribute to the progression of fibrotic changes by restricting mobility and facilitating pathological tissue remodeling. Additionally, a reduction in the thickness of both superficial and deep fasciae may further exacerbate these changes, leading to compromised fascial integrity and impaired gliding between tissue layers. This involvement could be crucial in understanding how fibrosis alters the biomechanical properties of the subcutaneous tissue, potentially impacting conditions such as lymphedema.

Our study also revealed statistically significant correlations between the thickness of forearm superficial fascia and BMI for the Ant 2 region in chronic lymphedema patients (r = −0.5135; *p* = 0.0001), while for forearm deep fascia is correlated with age for the Post 1 region in the chronic lymphedema patients (r = −0.2831; *p* = 0.0463). These findings underscore the likelihood of age- and BMI-dependent structural adaptations within fasciae in chronic lymphedema patients, reflecting a complex interplay between individual body composition factors and fascial integrity. The inverse correlation between BMI and the superficial fascia thickness suggests that an increased body mass may contribute to a thinning of superficial fascia, potentially due to mechanical stretching or alterations in connective tissue organization. In contrast, the age-related thinning observed in deep fascia could indicate a progressive loss of fascial elasticity or density, possibly driven by physiological aging processes that influence the collagen and elastin content [26,27,28,29]. Together, these variations reveal a layered and multifactorial relationship in which both body mass and age influence the physical properties of fasciae. This insight deepens our understanding of fascia behavior in chronic lymphedema, hinting at the need for personalized approaches in managing or mitigating fascia-related complications based on patient-specific factors like age and BMI.

### Limitations of Study and Future Perspectives

Larger, multi-centered studies are essential to validate these observations and provide a clearer understanding of tissue variability across diverse patient demographics. Moreover, as this research used a cross-sectional design, it cannot offer insights into the long-term progression or the impacts of different therapeutic interventions over time. A further limitation lies in the precision of US imaging in evaluating fasciae. Accurate assessment is highly contingent upon both skill level of the sonographer/clinician and the optimal calibration of the US equipment. These factors can introduce variability and potential bias in the findings. Therefore, standardized protocols, as employed in this study, and more comprehensive studies are required to bolster the consistency of the results and their translational value in clinical settings. In future classification systems for lymphedema, integrating US examination of fasciae could provide valuable precision, as demonstrated here. Considering the critical role of fasciae in lymphatic function, future investigation should involve larger patient cohorts. This approach would support a more comprehensive understanding of quantitative fascial changes. Moreover, future research should focus on systematically stratifying the US findings by lymphedema stage to establish stage-specific thresholds for fascial thickness. Integrating these findings with clinical markers such as fibrosis and functional impairment may pave the way for a more comprehensive, evidence-based classification framework. Finally, future research focusing also on the cellular and molecular mechanisms of the retinacula cutis’ contribution to fibrosis, as well as the implications of fascial thinning, could open up new therapeutic avenues that aim to target or modify these structures to mitigate fibrotic progression.

## 5. Conclusions

In conclusion, the present study highlights the value of US imaging as a non-invasive, effective tool for detecting subtle fascial alterations in chronic lymphedema patients. While no significant difference in fascial thickness was observed between affected and unaffected upper limbs, the notably thinner superficial and deep fasciae in lymphedema patients compared with healthy volunteers suggests a potential baseline alteration in these patients. This finding points toward a possible anatomical predisposition, where pre-existing fascial features may contribute to the development and persistence of chronic lymphedema. These insights underscore the importance of incorporating detailed US assessments in the diagnostic and management protocols for lymphedema, with the aim of identifying and monitoring fascial modifications that may otherwise go undetected. Such an approach could enhance early intervention strategies, improving outcomes and providing a deeper understanding of fascia’s role in lymphedema pathophysiology.

## Figures and Tables

**Table 1:** Descriptive data of the sample.

Data	Group 1	Group 2	*p*-Value Group 1 vs. Group 2
Age, y	58.43 ± 8.74	57.09 ± 7.38	*p* = 0.12
Weight, kg	70.22 ± 6.1	73.60 ± 8.20	*p* = 0.43
Height, cm	163.3 ± 4.83	168.30 ± 6.76	*p* = 0.52
BMI, Kg/cm^2^	26.35 ± 4.65	27.03 ± 6.1	*p* = 0.63

**Table 2:** Ultrasound thickness measurements of the arm superficial fascia at different levels and regions. Abbreviations: L = lymphedema.

Descriptive Statistics	Arm ANT 1 L	Arm ANT 2 L	Arm POST 1 L	Arm POST 2 L
Number of values	50	50	50	50
Minimum	0.17	0.2	0.19	0.25
Maximum	0.42	0.43	0.43	0.58
Range	0.25	0.23	0.24	0.33
Mean	0.2758	0.2838	0.2726	0.3466
Std. Deviation	0.04656	0.05774	0.06027	0.08424
Std. Error of Mean	0.006584	0.008166	0.008523	0.01191
Coefficient of variation	16.88%	20.35%	22.11%	24.30%

**Table 3:** Ultrasound thickness measurements of the arm superficial fascia at different regions and levels in the healthy volunteers.

Descriptive Statistics	Arm ANT 1	Arm ANT 2	Arm POST 1	Arm POST 2
Number of values	50	50	50	50
Minimum	0.2	0.21	0.24	0.24
Maximum	0.62	0.51	0.75	0.82
Range	0.42	0.3	0.51	0.58
Mean	0.3842	0.3816	0.5302	0.5208
Std. Deviation	0.09819	0.08402	0.1007	0.1071
Std. Error of Mean	0.01389	0.01188	0.01424	0.01515
Coefficient of variation	25.56%	22.02%	18.99%	20.57%

**Table 4:** Ultrasound thickness measurements of the arm superficial fascia at different levels and regions in the chronic lymphedema patients (group 1). Abbreviations: L = lymphedema.

Descriptive Statistics	Forearm ANT 1 L	Forearm ANT 2 L	Forearm POST 1 L	Forearm POST 2 L
Number of values	50	50	50	50
Minimum	0.18	0.17	0.22	0.18
Maximum	0.41	0.42	0.42	0.33
Range	0.23	0.25	0.2	0.15
Mean	0.2834	0.264	0.3164	0.2754
Std. Deviation	0.05278	0.04522	0.05506	0.03489
Std. Error of Mean	0.007465	0.006395	0.007787	0.004934
Coefficient of variation	18.63%	17.13%	17.40%	12.67%

**Table 5:** Ultrasound thickness measurements of the forearm superficial fascia at different regions and levels in the healthy volunteers (group 2).

Descriptive Statistics	Forearm ANT 1	Forearm ANT 2	Forearm POST 1	Forearm POST 2
Number of values	50	50	50	50
Minimum	0.2	0.2	0.17	0.25
Maximum	0.64	0.6	0.72	0.71
Range	0.44	0.4	0.55	0.46
Mean	0.3782	0.3558	0.3966	0.4356
Std. Deviation	0.09963	0.1028	0.1131	0.09324
Std. Error of Mean	0.01409	0.01454	0.01599	0.01319
Coefficient of variation	26.34%	28.89%	28.52%	21.41%

**Table 6:** Ultrasound thickness measurements of the deep fascia of the arm at the different levels and regions in the chronic lymphedema patients (group 1). Abbreviations: L = lymphedema.

Descriptive Statistics	Arm ANT 1 L	Arm ANT 2 L	Arm POST 1 L	Arm POST 2 L
Number of values	50	50	50	50
Minimum	0.31	0.3	0.25	0.38
Maximum	0.57	0.75	0.68	0.82
Range	0.26	0.45	0.43	0.44
Mean	0.4424	0.486	0.3856	0.5268
Std. Deviation	0.06005	0.1071	0.1037	0.122
Std. Error of Mean	0.008493	0.01515	0.01467	0.01725
Coefficient of variation	13.57%	22.04%	26.90%	23.16%

**Table 7:** Ultrasound thickness measurements of the deep fascia of the arm at different regions and levels in the healthy volunteers (group 2).

Arm ANT 1	Descriptive Statistics	Arm ANT 2	Arm POST 1	Arm POST 2
Number of values	50	50	50	50
Minimum	0.43	0.4	0.47	0.5
Maximum	1.11	0.9	1.2	1.26
Range	0.68	0.5	0.73	0.76
Mean	0.719	0.6464	0.7996	0.812
Std. Deviation	0.1408	0.1897	0.1372	0.1727
Std. Error of Mean	0.01558	0.01616	0.01941	0.02443
Coefficient of variation	18.35%	18.72%	17.16%	21.27%

**Table 8:** Ultrasound thickness measurements of forearm deep fascia at different levels and regions in the chronic lymphedema patients (group 1). Abbreviations: L = lymphedema.

Descriptive Statistics	Forearm ANT 1 L	Forearm ANT 2 L	Forearm POST 1 L	Forearm POST 2 L
Number of values	50	50	50	50
Minimum	0.22	0.25	0.28	0.26
Maximum	0.59	0.6	1.01	0.79
Range	0.37	0.35	0.73	0.53
Mean	0.4402	0.3922	0.536	0.4528
Std. Deviation	0.08494	0.0865	0.1498	0.116
Std. Error of Mean	0.01201	0.01223	0.02119	0.0164
Coefficient of variation	19.30%	22.06%	27.95%	25.61%

**Table 9:** Ultrasound thickness measurements of forearm deep fascia at the different regions and levels in the healthy volunteers (group 2).

Descriptive Statistics	Forearm ANT 1	Forearm ANT 2	Forearm POST 1	Forearm POST 2
Number of values	50	50	50	50
Minimum	0.44	0.34	0.49	0.051
Maximum	1.1	1.04	1.1	1.1
Range	0.66	0.7	0.61	1.049
Mean	0.68	0.6104	0.7034	0.7082
Std. Deviation	0.6004	0.1143	0.1209	0.204
Std. Error of Mean	0.01991	0.02683	0.0171	0.02885
Coefficient of variation	19.58%	29.35%	17.19%	28.81%

## Data Availability

The data presented in this study are available upon request from the corresponding author. The data are not publicly available due to privacy considerations.
